# Retraction for Polev et al., “Draft Genome Sequence of Geotrichum candidum Strain 3C”

**DOI:** 10.1128/MRA.00561-19

**Published:** 2019-06-13

**Authors:** Dmitrii E. Polev, Kirill S. Bobrov, Elena V. Eneyskaya, Anna A. Kulminskaya

**Affiliations:** a Research Resource Center for Molecular and Cell Technologies, St. Petersburg State University, St. Petersburg, Russia; b National Research Centre, Kurchatov Institute, B.P. Konstantinov Petersburg Nuclear Physics Institute, Gatchina, Orlova Roscha, Russia; c St. Petersburg State Polytechnical University, St. Petersburg, Russia

## RETRACTION

Volume 2, no. 5, e00956-14, 2014, https://doi.org/10.1128/genomeA.00956-14. We retract this article. The Geotrichum candidum strain 3C was initially identified and classified in the early 1970s (N. A. Rodionova, N. A. Tiunova, R. V. Feniksova, T. I. Kudriashova, and L. I. Martinovich, Dokl Akad Nauk SSSR 214:1206–1209, 1974). In 2014, we had sequenced and published its draft genome, so the genome analysis of the strain became possible (D. E. Polev, K. S. Bobrov, E. V. Eneyskaya, and A. A. Kulminskaya, Genome Announc 2:e00956-14, 2014, https://doi.org/10.1128/genomeA.00956-14). After the publication of the draft genome sequence of the G. candidum strain CLIB 918 (ATCC 204307) in 2015 (G. Morel, L. Sterck, D. Swennen, M. Marcet-Houben, et al., Sci Rep 5:11571, 2015, https://doi.org/10.1038/srep11571 [Erratum, 5:12596, https://doi.org/10.1038/srep12596]) and due to the inconsistency between these genome sequences, it became clear that G. candidum strain 3C needed to be reclassified. In further work, the yeast-like fungus previously known as G. candidum 3C was reclassified as Scytalidium candidum 3C (I. Y. Pavlov, K. S. Bobrov, A. D. Sumacheva, A. E. Masharsky, et al., J Basic Microbiol 58:883–891, 2018, https://doi.org/10.1002/jobm.201800066).

We apologize to the readers of *Genome Announcements*/*Microbiology Resource Announcements* for any inconvenience that this causes.

